# Who Will Be My Friend? The Role of the Liking Gap in Preschooler Friend Selection

**DOI:** 10.3390/bs15020196

**Published:** 2025-02-12

**Authors:** Jialu Liu, Kainian Mo, Zhiqiang Yan, Xiao Zeng

**Affiliations:** 1 School of Education, Hunan First Normal University, Changsha 410205, China; l13627323101@icloud.com (J.L.); dccexx26@163.com (K.M.); 2 College of Educational Science, Hengyang Normal University, Hengyang 421010, China; 3 Department of Psychology, Hunan Normal University, Changsha 410081, China; yanzhiqiangpsy@hunnu.edu.cn

**Keywords:** egocentric, friend selection, liking gap, peers, perspective-taking, preschoolers

## Abstract

Friend selection is a crucial aspect of social development, particularly during preschool years. This study investigated the role of the liking gap in shaping preschoolers’ friend selection mechanisms through two experiments. In Experiment 1 (*N* = 120), a liking gap task was used to examine whether preschoolers perceive a discrepancy between how much they like familiar friends and how much they believe those friends like them in return. The results indicated that preschoolers tend to assume that their most liked peers evaluate them less positively than they do in return, whereas they believe their least liked peers evaluate them more favorably than expected. In Experiment 2 (*N* = 117), a friend selection task was conducted to assess whether the liking gap influences preschoolers’ choices of friends among unfamiliar peers. The findings revealed that while preschoolers prefer to befriend individuals they like or who like them, their decisions also reflect an awareness of how they are perceived by others. These results suggest that by ages 5 to 6, preschoolers develop an emerging sensitivity to social evaluations, which plays a role in their friend selection process.

## 1. Introduction

Preschoolers’ interactions with peers play a pivotal role in their social and emotional development, yet the underlying mechanisms guiding their friend selection remain an area warranting further investigation. During early childhood, preschoolers spend substantial time socializing and playing with peers (Kenneth et al., 2009), which provides opportunities to develop essential social skills and cultivate positive emotions (Daniels et al., 2010; Rabaglietti et al., 2012). Through these interactions, they learn to navigate social norms, negotiate rules, and resolve disagreements, all of which contribute to their social competence (Danby et al., 2012). Moreover, peer relationships are linked to preschoolers’ mental health, underscoring their broader developmental significance (X. Wang et al., 2024).

The importance of peer relationships has long been recognized in developmental theory. Piaget and Inhelder (1969) argued that peer relationships are more egalitarian than parent–child relationships, fostering mutual respect and understanding. Sullivan (1940) elaborated on this, emphasizing the role of peer interactions in shaping interpersonal skills and emotional growth. Preschoolers not only learn how to get along with others but also form close friendships through peer interactions (Mcdonald & Rubin, 2012). Prior research highlights a significant association between peer relationships and happiness in preschoolers (Holder & Coleman, 2007), further illustrating their relevance to both current well-being and future development.

Given the profound influence of peer relationships on preschoolers’ development, understanding the factors that shape peer selection is crucial. Investigating the processes underlying friend selection can provide insights into the dynamics of preschoolers’ peer relationships and inform strategies to foster positive and supportive friendships.

### 1.1. Liking Gap

Many factors influence preschoolers’ mechanisms of friend selection, one of which is the liking gap. It is often difficult for individuals to accurately gauge how their partners truly perceive them, leading to uncertainty about how much others like them, enjoy their company, and desire future interactions (Boothby et al., 2018). Prior research has conceptualized the liking gap as a form of negative social bias—an illusion in which individuals tend to underestimate how much others like them following brief interactions (Mastroianni et al., 2021). Boothby et al. (2018) first identified this phenomenon, observing that people systematically undervalue how much their interaction partners enjoy their company. For instance, an individual might assume their partner rates their liking as a 3 on a scale, whereas the actual rating might be a 7. While individuals often hold unrealistically positive views of themselves and their abilities (Alicke, 1985; Kruger & Dunning, 1999), this optimism does not extend to their perceptions of social life. People frequently believe others have more active and fulfilling social lives than they do (Deri et al., 2017; Zhu et al., 2023), maintain more frequent contact with others (Whillans et al., 2017), and desire less contact with them than vice versa (Epley & Schroeder, 2014). Wolf et al. (2021) extended the concept of the liking gap to children aged 4–11, focusing on interactions between unfamiliar peers. Their findings suggest that the liking gap begins to emerge around the age of 5, coinciding with children’s increasing awareness of how they are perceived by others. Thus, the ages of 5 to 6 represent a critical developmental stage when children start to actively consider others’ perspectives in social interactions.

However, existing research on the liking gap has notable limitations. Most studies, including Boothby et al. (2018) and Wolf et al. (2021), focus on interactions between strangers and rely on brief encounters followed by evaluations of mutual liking. While Wolf et al. (2021) examined the actual liking gap—the discrepancy between perceived and actual levels of liking—they did not investigate the hypothetical liking gap, which is based solely on individuals’ assumptions. Moreover, previous research has largely overlooked the underlying mechanisms that contribute to this psychological gap, leaving gaps in our understanding of the phenomenon in young children. Our study aims to address these limitations by examining preschoolers’ assumptions about others’ evaluations, focusing on their self-perceived liking gap.

Importantly, previous studies have not explored whether the liking gap exists in established peer relationships, such as friendships, or how it influences friend selection. Unlike earlier work that primarily examined interactions with strangers, our study investigates whether the liking gap impacts preschoolers’ decisions about choosing friends, particularly after they learn about their peers’ level of liking for them. In this context, we conceptualize the liking gap as the difference between the degree to which preschoolers like others and the degree to which they assume others like them. Our study specifically focuses on preschoolers’ internal perceptions, providing a framework to explore the factors contributing to this psychological gap and its implications for their friend selection processes.

### 1.2. The Reasons Why the Liking Gap Can Affect Friend Selection

Several reasons suggest that the liking gap may influence preschoolers’ friend selection.

First, preschoolers exhibit a self-centered approach to friend selection, favoring individuals who fulfill their emotional needs. Piaget and Inhelder (1969) posited that children aged 5 to 6, who are in the preoperational stage of development, display egocentric tendencies. According to egocentricity theory, preschoolers primarily base their friend selection on their personal preferences. Cassidy et al. (2003) observed that young children often choose as friends those they like most and wish to spend time with. Additionally, Liberman and Shaw (2019) noted that children are more likely to select friends who meet their emotional needs, reflecting an active preference for peers they find emotionally satisfying. This self-directed inclination underscores the role of personal liking in preschoolers’ friend selection.

Second, the social influence of peer groups plays a significant role in preschoolers’ friend selection. Bronfenbrenner’s (1974) ecological systems theory highlights the importance of the microsystem—the immediate environments in which children interact directly, such as peer groups. At ages 5–6, the microsystem exerts the most substantial influence on children’s early development (Bronfenbrenner, 1994). Peer groups within this microsystem often have a more profound impact on preschoolers’ socialization than other groups, including parents and teachers (Jensen, 2018). Through increased interactions with peers, preschoolers gradually begin to consider others’ perspectives and evaluations when selecting friends (Gottman, 1983). This growing awareness of others’ viewpoints suggests that preschoolers are influenced not only by their own preferences but also by the perceived opinions of their peers.

### 1.3. Current Studies

Wolf et al. (2021) demonstrated the presence of the liking gap among strangers but did not investigate its occurrence in more familiar social contexts, such as among classmates. Although prior studies have extensively examined the liking gap (Boothby et al., 2018; Wolf et al., 2021), they primarily focus on actual perceived liking and do not explored children’s imaginative thinking or the underlying psychological mechanisms contributing to this phenomenon. To address these gaps, this study aims to determine whether the liking gap exists among preschoolers and their classmates, thereby broadening the scope of existing research.

This study seeks to provide valuable insights into preschoolers’ internal thoughts during peer interactions and to elucidate the mechanisms underlying their friend selection processes. Unlike previous studies, which have predominantly examined the liking gap in stranger interactions, our study investigates its presence in a classroom context. Experiment 1 shifts the context from strangers to classmates to determine whether the liking gap is present in familiar peer interactions. Using a peer nomination task and a liking gap task, we examined preschoolers’ perceptions of how much they like their classmates versus how much they believe their classmates like them. This experiment tests the following hypothesis:

**H1.**  *When interacting with familiar friends, preschoolers exhibit a liking gap.*

Building on the findings of Experiment 1, Experiment 2 explores whether knowledge of how much others like them influences preschoolers’ friend selection. By creating four scenarios that vary in the degree of liking, this experiment examines the rules governing friend selection when the liking gap is present. A friend selection task was used to assess the impact of the liking gap on friend selection. We propose the following hypothesis:

**H2.**  *When interacting with unfamiliar peers, preschoolers consider whether others like them during the process of friend selection.*

## 2. Experiment 1: Does the Liking Gap Exist in the Friend Selection Process of Preschooler?

It remains unclear whether the liking gap exists among classmates. To address this, Experiment 1 first assessed participants’ peer nominations, followed by questions designed to measure the liking gap. In this task, participants evaluated how much they liked their peers (self-assessment) and how much they believed their peers liked them (other-assessment).

### 2.1. Participants

After obtaining active parental consent, 120 children (63 girls, 57 boys) from a public kindergarten in Changsha, China, participated in the study. The participants’ average age was 67.32 months (*SD* = 3.53). A G*Power v. 3.1.9.2. analysis was conducted to determine the required sample size for a 2 × 2 repeated measures ANOVA with two levels per group. Assuming a medium effect size (*f* = 0.25), a significance level of 0.05, and a desired power of 0.95, the analysis indicated that the sample size in the current study is adequate to meet these criteria. The study was approved by the Ethics Committee of our university, and all contacted families agreed to participate, signing a written informed consent form to authorize their children’s involvement.

### 2.2. Procedures

The experiment began by obtaining verbal assent from the preschoolers. If they agreed to participate, researchers assessed peer relationships using the peer nomination method, followed by the liking gap task. Upon completing the tasks, participants received a small gift as a token of appreciation.

### 2.3. Measures

Experiment 1 utilized a 2 (Peers: Like the Most, Like the Least) × 2 (Perspectives: Self, Other) within-subject experimental design to examine whether preschoolers’ friend selection is influenced by the liking gap.

#### 2.3.1. The Peer Nomination Task

Preschoolers completed a peer nomination task to identify classmates they liked the most and the least (Kurdek & Rodgon, 1975). Participants were provided with a class roster and asked to circle at least three names of classmates they liked the most and three they liked the least.

#### 2.3.2. The Liking Gap Task

**Figure 1:**
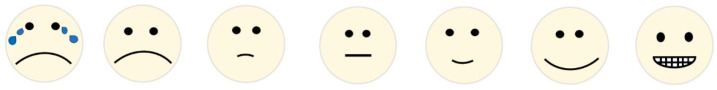
Seven-point smiley-face Likert-type scale.

The liking gap task was performed to assess how much they like others and how much they think others like them. Researchers selected the first “most liked” and “least liked” names circled during the peer nomination task and asked each preschooler twice, and six questions each time, to verify the existence of a liking gap. To ensure comprehension of the task and 7-point smiley-face Likert-type scale (See Figure 1), two practice questions were administered (e.g., “Do you like ice cream? How much do you like ice cream?”) following Wolf et al. (2021). Participants who did not respond appropriately were excluded from subsequent analyses. The six questions directly referenced Wolf’s experimental paradigm. Participants then answered six questions divided into two sets of three. Self-perspective questions: (1) How much do you like him/her? (2) How much do you want to play games with him/her? (3) How much do you want him/her to be your friend? Other-perspective questions: (1) How much do you think others like you? (2) How much do you think others want to play games with you? (3) How much do you think others want to be your friends? The questions were presented in a counterbalanced order. The average scores for each participant’s responses in the self- and other-perspective were calculated.

### 2.4. Analytic Plan

Data were analyzed using Jamovi software v. 2.3.28.0. A repeated-measures ANOVA was conducted to test whether preschoolers’ responses differed significantly between the “most liked” and “least liked” peers across self- and other-perspectives. This approach allowed for the evaluation of within-subject differences in scores across the experimental conditions.

### 2.5. Results

Descriptive statistics indicated that the data met the normality criteria outlined by established research guidelines (skewness < 2; kurtosis < 7, West et al., 1995), making it suitable for subsequent analysis.

A 2 (Peers: Like the most, Like the least) × 2 (Perspective: Self, Other) repeated-measures ANOVA was employed. The data in Table 1 are composed of the mean and standard deviation of the liking scores of preschoolers regarding the classmates they like the most and the classmates they like the least. The liking scores were derived by averaging each participant’s responses to the six questions, considering both self- and other-perspectives. It comprises two sets of data: one is the score of how much you like others, the other is the score of how much you think others like you.

ANOVA results showed us there are two main effects: peers [*F*(1,119) = 420.20, *p* < 0.001, $\eta_{P}^{2}$ = 0.78] and perspectives [*F*(1,119) = 4.73, *p* = 0.03, $\eta_{P}^{2}$ = 0.04]. Follow-up pairwise comparisons showed that preschoolers think that they evaluate the classmate they like the most more positively than the classmate they like the most evaluates them (*t*(119) = 20.50, *p* < 0.001). Additionally, preschoolers think that they evaluate the classmate they like the least more negatively than the classmate they like the least evaluates them (*t*(119) = −2.18, *p* = 0.03). The results indicated that a liking gap emerges when preschoolers interact with familiar friends.

A significant interaction between peers and perspectives was also observed [*F*(1,119) = 110.09, *p* < 0.001, $\eta_{P}^{2}$ = 0.48]. Follow-up pairwise comparisons (all pairwise comparisons in the current article were performed using the Bonferroni correction) showed that a liking gap existed between different peers. Preschoolers think that friends evaluate them (*M* = 5.53, *SE* = 0.12) less positively than they evaluate their friends (*M* = 6.25, *SE* = 0.08), specifically the classmate they like the most, and they think that they evaluate the classmate they like the least (*M* = 2.22, *SE* = 0.11) less positively than the classmate they like the least evaluates them (*M* = 3.29, *SE* = 0.15).

### 2.6. Discussion

The findings from Experiment 1 demonstrate a liking gap among preschool classmates. When interacting with their most-liked peers, preschoolers perceive that their peers evaluate them less positively than they evaluate these peers. Similarly, preschoolers also believe they evaluate their least-liked peers less favorably.

These results align with Boothby et al.’s (2018) findings but reveal unique age-related nuances. First, preschoolers’ preference for selecting their most-liked peers as friends can be explained through Piaget’s (1928) theory of egocentrism, which posits that preschoolers view the world from their own perspective and struggle to consider alternative viewpoints. This preference reflects the developmental characteristics of children in the preoperational stage.

Second, the liking gap was evident among 5- to 6-year-old children. Preschoolers believe that the classmates they like most reciprocate their affection, but they tend to underestimate the extent of this reciprocity. As cognitive abilities advance, Bandura’s (1989) social cognitive theory suggests that children increasingly acquire information by observing others, developing schemas about social relationships. By around age five, preschoolers begin to show concern for reputation (Engelmann & Rapp, 2018), seeking to manage how others perceive them. This developmental mechanism may lead preschoolers to believe that others’ evaluations of them are less favorable than their actual self-appraisals.

The presence of a liking gap in preschoolers’ interactions with friends suggests that their friend selection is primarily influenced by their own preferences. First, preschoolers are more likely to choose friends who resemble themselves (Laursen, 2017), as their preferences are centered on their own experiences. Second, they gravitate toward peers with positive traits that benefit them, such as friendliness and helpfulness (Piaget & Inhelder, 1969; Y. L. Wang et al., 2021). Third, forming friendships with peers they like evokes positive emotions, which play a critical role in fostering peer relationships and shaping the tone of social interactions (Hernández et al., 2017; Moreira et al., 2021).

However, egocentrism represents only one aspect of preschoolers’ development, which is a dynamic and multifaceted process. Bandura’s (1989) theory highlights that children also engage in observing and interpreting others’ behaviors during social interactions. They gradually recognize that positive behaviors from others may partly serve self-oriented goals, such as reputation management. Cognitive theories further suggest that these expectations influence how preschoolers perceive and interpret social interactions, often leading to the overestimated evaluations of others’ behaviors (Hui et al., 2020). Additionally, Bronfenbrenner’s (1974) ecological systems theory emphasizes the impact of various social environments on children’s development. These contexts shape both the presence of the liking gap and the fluid nature of friend selection among preschoolers.

## 3. Experiment 2: The Impact of the Liking Gap on Preschoolers’ Friend Selection

Building on the findings from Experiment 1, which confirmed the existence of a liking gap through a validated task, Experiment 2 aimed to explore whether preschoolers’ friend selection is influenced by this phenomenon. Unlike Experiment 1, which focused on familiar classmates, Experiment 2 examined interactions with strangers. Additionally, Experiment 2 introduced two distinct liking conditions—Like–Dislike and Dislike–Like—to further examine the characteristics of preschoolers’ friend selection. Building on Boothby et al. (2018), we incorporated a total of four distinct liking gap scenarios to explore how this gap influences the mechanisms underlying preschoolers’ friend selection.

### 3.1. Participants

Participants were 117 children (60 girls, 57 boys) who did not take part in Experiment 1. All participants were recruited from four different classes in a public kindergarten in the Changsha region, China. Their mean age was 66.29 months (*SD* = 3.14). Based on the G*Power analysis, the current study’s sample size is sufficient to detect significant effects under the specified conditions. Written informed consent was obtained from parents before data collection, and ethical approval was granted by the university’s Ethics Committee. All contacted families agreed to participate, and children were informed that they could withdraw from the study at any time without providing a reason.

### 3.2. Procedures

Researchers began by obtaining verbal consent from the children to participate in the experiment. Participants then engaged in a friend selection task presented on a tablet. The task included four story-based scenarios, each followed by a 7-point smiley-face Likert-type scale (See Figure 1) for response. After completing the task, children received a small gift as a token of appreciation.

### 3.3. Measures

Experiment 2 utilized a 2 (Perspectives: Self, Other) × 4 (Situations: Like–Dislike, Dislike–Like, Dislike–Dislike, Like–Like) within-subject design. The task consisted of four stranger-based scenarios that varied in the degree of the liking gap. For instance, in the Like–Dislike condition, “Like” indicated that the child liked the stranger, while “Dislike” indicated that the stranger did not like the child.

Scenarios were adapted from prior research (Cassidy et al., 2003; Chernyak & Kushnir, 2013) within the framework of social cognition. The Like–Dislike and Dislike–Like conditions reflected the traditional liking gap paradigm, while the Like–Like and Dislike–Dislike conditions served as control conditions. A pre-experiment ensured that participants could comprehend the scenarios and respond appropriately. By assessing both the participant’s feelings towards the stranger and their perception of the stranger’s feelings towards them, this design allows for a comprehensive understanding of the preschooler’s social cognitive abilities in the context of interpersonal relationships. Experiment 2 comprised four liking gap situations: Like–Dislike (You really like her/him, but she/he does not like you), Dislike–Like (You do not like her/him, but she/he really likes you), Dislike–Dislike (You do not like her/him, and she/he does not like you), and Like–Like (You really like her/him, and she/he also really likes you).

For example, Situation A describes a Like–Dislike scenario. In this scenario, participants were told, “An unfamiliar child has transferred to your class. You really like her/him, but she/he does not like you.” After the participants understood this scenario, the experimenter asked them four questions: (1) Do you want to be friends with her/him? (2) How much do you want/do you not want to be friends with her/him? (3) Do you think she/he wants to be friends with you? (4) How much does she/he want/not want to be friends with you? After each question, the experimenter presented a seven-point smiley-face Likert-type scale (see Figure 1), which was the same as in Experiment 1. Participants were required to point to and name a specific face, and the experimenter recorded the scores, ranging from 1 (least positive) to 7 (most positive) (see Figure 1). The first two questions evaluate the self-perspective, while the last two focus on the other-perspective.

### 3.4. Analytic Plan

Data analysis was conducted using Jamovi software. A repeated-measures ANOVA was performed to examine the impact of different perspectives and scenarios on friend selection. This approach enabled the identification of significant differences in scores across varying conditions.

### 3.5. Results

Descriptive statistics indicated that the data met the normality criteria outlined by established research guidelines (skewness < 2; kurtosis < 7, West et al., 1995), making it suitable for subsequent analysis.

A 2 (Perspectives: Self, Other) × 4 (Situations: Like–Dislike, Dislike–Like, Dislike–Dislike, Like–Like) repeated-measures ANOVA was employed. The data in Table 2 are composed of the mean and standard deviation of the scores of how much you want to be friends with the stranger and the scores of how much you think the stranger wants to be friends with you. It includes preschoolers’ scores in four different story situations.

The ANOVA results of Experiment 2 showed a main effect of situations [*F*(3,348) = 91.22, *p* < 0.001, $\eta_{P}^{2}$ = 0.44]. Follow-up pairwise comparisons showed that the liking score of the situation of Like–Like was the highest (*p*s < 0.001). First, comparing Like–Like with Dislike–Like (*t*(116) = −8.03, *p* < 0.001), we identified that preschoolers think more about whether they like each other or not when choosing their friends. Second, comparing Dislike–Dislike with Dislike–Like (*t*(116) = 9.02, *p* < 0.001), we identified that preschoolers also consider whether others like them or not when choosing their friends. However, we did not find the main effect of perspectives [*F*(1,116) = 0.06, *p* = 0.80, $\eta_{P}^{2}$ < 0.01]. The results revealed no main effect of perspective, indicating that preschooler’s responses reflecting their own feelings (self-perspective) and their perceptions of the stranger’s feelings (other-perspective) were statistically comparable across all scenarios. This finding suggests that preschoolers employ similar evaluative processes when considering their own preferences and inferring the preferences of others.

A significant interaction between perspectives and situations was identified [*F*(3,348) = 40.50, *p* < 0.001, $\eta_{P}^{2}$ = 0.26]. Follow-up pairwise comparisons revealed that liking scores influenced preschoolers’ friend selection mechanisms. From the self-perspective, preschoolers considered themselves more likely to choose friends in the Like–Like situation and less likely in the Dislike–Dislike situation. From the other-perspective, preschoolers believed that others were more likely to choose them as friends in the Like–Like and Dislike–Like situations, and less likely in the Like–Dislike and Dislike–Dislike situations.

### 3.6. Discussion

The findings of Experiment 2 indicate that preschoolers are more inclined to select as friends those they personally like, consistent with the results of Experiment 1. However, in situations involving strangers, preschoolers also showed a greater tendency to choose as friends those who expressed liking for them.

This tendency to focus on others’ evaluations in stranger situations can be attributed to several developmental factors. First, preschoolers gradually transition from egocentrism to perspective-taking as their cognitive abilities develop. Research by Wellman et al. (2001) demonstrates that by around the age of four, children acquire a representational theory of mind, enabling them to infer and reason about others’ mental states, including abstract constructs such as beliefs and false beliefs. Preschoolers can predict the emotions of others and provide feedback (Shi et al., 2024). As preschoolers age, their mental understanding becomes more sophisticated, a process facilitated by increased opportunities for cooperative social interactions and exposure to diverse psychological states (Slaughter et al., 2010). Bronfenbrenner’s (1974) ecological systems theory underscores the influence of environmental factors on development. Consistent with this framework, preschoolers’ increasing social interactions foster awareness of others’ perspectives and evaluations, reflecting their gradual decentralization from egocentric thinking. This finding aligns with Boothby et al. (2018), who also highlighted the role of social experiences in advancing perspective-taking abilities. As preschoolers engage more frequently in social interactions, they become increasingly conscious of how others perceive them (Baird & Grace, 2021).

Second, the preschoolers’ attention to others’ liking can also be understood through Maslow’s hierarchy of needs, specifically the need for belongingness and love. This need reflects an innate desire to form emotional connections and receive understanding, care, and recognition (Maslow, 1954). The need for companionship, friendship, and partnership is particularly salient among preschoolers aged 5–6 (Ma, 2013), as emotional and intimate relationships with peers provide critical social experiences necessary for growth.

Third, as preschoolers increasingly focus on others’ evaluations, they may encounter social anxieties inherent to interpersonal interactions. This heightened sensitivity can increase the salience of negative thoughts and lead to skepticism regarding others’ perceptions of them (Wolf et al., 2021). While preschoolers often maintain optimism about their abilities across various domains, this confidence does not extend to social interactions (Alicke, 1985).

When encountering strangers, preschoolers’ lack of familiarity may amplify their reliance on external evaluations. The liking gap, a phenomenon driven by individuals’ anxieties about how they are perceived during interactions (Boothby et al., 2018), further emphasizes the significance of others’ evaluations in friend selection. Preschoolers may, in some situations, express a willingness to befriend individuals they personally dislike. This could be driven by a desire to gain a sense of belonging and avoid social isolation or to obtain strategic benefits such as resources or protection (Cacioppo et al., 2009). Additionally, when others initiate cooperation or demonstrate prosocial behavior, preschoolers may reciprocate this gesture by expressing willingness to be friends, even with peers they dislike (Tomada & Schneider, 1997). In the “Dislike–Like” scenario, the expressed desire of the disliked peer to form a friendship may significantly influence the preschoolers’ responses.

## 4. General Discussion

The present experiment highlights the significant role the liking gap plays in motivating preschoolers’ friend selection. In Experiment 1, when interacting with familiar friends, preschoolers exhibited a liking gap. These findings suggest that while preschoolers are still in a developmental stage characterized by egocentrism, their friend selection also reflects an emerging awareness of others’ perspectives. In Experiment 2, when placed in scenarios involving strangers, preschoolers were more inclined to select others as friends when a liking gap was present. The findings from both experiments provide valuable insights into the process of friend selection among preschoolers. They reveal that although preschoolers exhibit egocentric tendencies in their friend selection, they also begin to engage in perspective-taking.

Drawing on the previous literature and theoretical frameworks, our analysis contributes to the understanding of social interactions and peer relationships among preschoolers. From the perspective of social ecosystem theory, peer groups play a pivotal role in shaping preschoolers’ social development. Within microsystems, preschoolers interact directly with peers, forming meaningful social connections (Bronfenbrenner, 1994). Friends significantly influence these interactions, shaping preschoolers’ social experiences and overall development (Jensen, 2018).

Consistent with Piaget and Inhelder’s (1969) work, preschoolers demonstrate a strong tendency to prioritize their personal preferences when choosing friends. At the preoperational stage, egocentrism remains a dominant feature of cognitive development, leading children to perceive themselves as central to their social world. As a result, their friend selection is often shaped by their own subjective likes and dislikes (Choifer, 2021). However, preschoolers aged 5 to 6 years also exhibit developmental advancements, as evidenced by their growing ability to consider the perspectives of others (Wolf et al., 2021). This shift suggests that they are beginning to move beyond purely egocentric reasoning and are developing a more nuanced understanding of social interactions.

When preschoolers recognize the existence of a liking gap in relationships, they are more likely to choose both they like and those who like them as friends. This suggests that in interactions with others, preschoolers’ friend selection is increasingly influenced by concerns about how they are evaluated by others (Wolf et al., 2021). This progression indicates that preschoolers are beginning to utilize a second-order theory of mind, enabling them to process more complex social information (Carpendale & Lewis, 2004; Tomasello et al., 2005).

These findings align with broader themes in social development, particularly the emergence of peer relationships among preschoolers. The role of the liking gap in guiding social interactions represents a crucial developmental milestone, laying the foundation for the acquisition of more advanced social skills later in life. This study adds to the growing body of research emphasizing both the cognitive limitations and the influence of the liking gap on friend selection in early childhood. It marks an important step in deepening our understanding of the complexities of social development and friendship formation during this formative period.

### Limitations and Future Research

Future research can address several areas. First, it is unclear whether the effect of the liking gap on friend selection lasts over time. Future studies could explore whether this effect continues in children over the age of 6 and how it influences friend selection at different stages. Additionally, future research will increase its focus on longitudinal studies to better understand age differences in the liking gap, examining how this phenomenon evolves across various developmental periods. Second, the current study used self-reported data, which may have limitations. Future research should use additional methods, such as behavioral observations or peer reports, to gather more accurate information. Third, other factors that may affect the liking gap, such as parents’ education, cultural background, economic conditions, and gender differences, should also be considered. Investigating how these factors influence the liking gap among preschoolers could provide valuable insights into how social relationships develop. Fourth, this study focused on preschoolers’ preferences and their perceptions of others’ preferences towards them but did not measure the actual preferences of the classmates. Future research could address this limitation by investigating the actual reciprocity of liking between participants and their peers.

## 5. Conclusions

In conclusion, the present study underscores the existence of a liking gap in preschoolers’ interactions with both familiar friends and strangers. When interacting with familiar friends, preschoolers exhibited a clear liking gap, tending to underestimate how much others like them. Moreover, when selecting friends among unfamiliar peers, their choices were influenced not only by their own preferences but also by the preferences of others. These findings suggest that preschoolers are beginning to integrate others’ evaluations into their friend selection process, with their decisions increasingly shaped by social dynamics. This reflects an emerging capacity for perspective-taking, as preschoolers become more attuned to and responsive to others’ preferences and evaluations when forming social relationships. 

## Figures and Tables

**Table 1:** Descriptive statistics of Experiment 1 (*N* = 120).

	The Classmate They Like the Most			The Classmate They Like the Least		
	*M/SD*	Skewness	Kurtosis	*M/SD*	Skewness	Kurtosis
Self-Perspective	6.25/0.86	−2.07	5.56	2.22	1.28	1.84
Other-Perspective	5.53/1.27	−1.09	0.63	3.29	0.66	−0.46

Note: Self-Perspective: the participants’ feelings toward their classmate; Other-Perspective: the participants thought their classmate felt about them.

**Table 2:** Descriptive statistics of Experiment 2 (*N* = 117).

	Self-Perspective			Other-Perspective		
	*M/SD*	Skewness	Kurtosis	*M/SD*	Skewness	Kurtosis
Like–Dislike	4.94/2.25	−0.72	−1.06	3.78/2.12	0.05	−1.45
Dislike–Like	4.75/2.19	−0.55	−1.18	6.07/1.34	−1.99	4.42
Dislike–Dislike	3.74/2.03	0.01	−1.28	3.80/2.10	0.03	−1.32
Like–Like	6.55/1.07	−1.38	5.96	6.40/1.19	−1.05	3.04

Note: Self-Perspective: the participants’ feelings toward their classmate; Other-Perspective: what the participants thought their classmate felt about them.

## Data Availability

The raw data supporting the conclusions of this article will be made available by the authors on request.
